# Low-Dose Radiation for Knee Osteoarthritis: A Case Report

**DOI:** 10.7759/cureus.107955

**Published:** 2026-04-29

**Authors:** Molly R Manning, Thomas R Eanelli

**Affiliations:** 1 Radiation Oncology, Touro College of Osteopathic Medicine, Middletown, USA; 2 Radiation Oncology, Garnet Health, Middletown, USA

**Keywords:** arthritis clinic, complementary and alternative therapy, knee osteoarthritis, low-dose radiation therapy, multidisciplinary approach

## Abstract

Traditional osteoarthritis (OA) treatment focuses on symptom management, including nonsteroidal anti-inflammatory drugs (NSAIDs), duloxetine, intra-articular glucocorticoids, or surgical intervention. Unfortunately, these approaches may provide inadequate symptom relief or can be contraindicated in patients with comorbidities such as cardiac issues, diabetes, and liver dysfunction. This underscores the necessity for alternative management options for OA. Low-dose radiation therapy (LDRT), widely used in European countries for the management of painful musculoskeletal conditions, has emerged as a promising option for pain relief in OA. We present the case of a 72-year-old man with a history of seven meniscectomies and bilateral knee OA. Due to minimal pain relief from prior treatments, the patient received 50 cGy per fraction (total of 3 Gy/6 fractions) to each knee three times per week for two weeks. After the completion of this treatment, the patient experienced substantial pain relief, meeting the Osteoarthritis Research Society International (OARSI) responder criteria. This case aims to discuss the need to optimize OA management by establishing collaborative, multidisciplinary clinics within hospitals.

## Introduction

Osteoarthritis (OA) is a degenerative joint disease characterized by cartilage degradation, bone remodeling, osteophyte formation, and joint inflammation [1]. OA is a major contributor to disability in older adults, impacting over 500 million individuals worldwide in 2019, with the knee being the most commonly affected joint, accounting for 60.6% of prevalent cases [2]. OA affects a third of people over the age of 70 and is ranked in the top 10 leading causes of years lived with disability in this age group [3]. OA management focuses on minimizing pain and making lifestyle changes [1]. Non-pharmacological interventions are first-line treatments, including exercise, weight loss, and knee braces, followed by pharmacological therapy, such as oral and topical nonsteroidal anti-inflammatory drugs (NSAIDs). Unfortunately, prolonged use of NSAIDs has been associated with cardiovascular, renal, and gastrointestinal side effects [4]. In a systematic review and meta-analysis of 192 randomized trials of NSAID and opioid interventions for knee and hip OA, an increased risk of any adverse event was observed for 14 of the 47 NSAIDs assessed [5]. In addition, a recent prospective cohort study of 4,197 individuals with knee or hip OA found that long-term NSAID use was associated with greater odds of clinically meaningful increases in pain (OR=2.04; 95% CI: 1.66 to 2.49), disability (OR=2.21; 95% CI: 1.74 to 2.80), and stiffness (OR=1.58; 95% CI: 1.29 to 1.93), compared to no long-term NSAID use over 4-5 years of follow-up [6]. While the authors noted the potential for bidirectional causality, this study does provide evidence for an association between long-term NSAID use and exacerbating symptoms.

Surgical interventions are also used to manage pain and improve function for severe OA. Although procedures such as total joint arthroplasty (TJA) are effective in relieving pain and restoring function, they are associated with risks, including surgical site infection, deep vein thrombosis (DVT), and implant failure, which may necessitate revision surgery and increase healthcare costs [7]. Implant failure, often accompanied by pain, functional impairment, and inflammation, is a leading indication for TJA revision. Moreover, compared with primary procedures, revision surgeries are technically more demanding, including managing bone loss and ensuring stable fixation and alignment. Prosthetic joint infections account for approximately 21.6% of TJA revisions and are often difficult to treat. Additionally, adherence to postoperative physical therapy can be challenging because of logistical barriers and patient motivation [8].

The physical limitations and pain caused by OA are not always adequately addressed with conservative management and pharmacological therapy. Surgery may be unacceptable or unsuitable for some patients with severe OA, given the associated risks and the requirement for postoperative rehabilitation [7]. One alternative pain management approach is low-dose radiation therapy (LDRT), which involves the use of low-dose radiation to reduce inflammation [4]. In the context of OA, LDRT typically is fraction sizes of 0.5-1.0 Gy per fraction, administered two to three times per week, delivered to a total dose of 3-6 Gy per treatment course. Although the mechanisms remain under investigation, research suggests that LDRT suppresses immune cells, thereby reducing joint inflammation and providing pain relief. LDRT has been used to treat OA for decades and was used in the United States until the 1980s when new pharmacological approaches became available. However, LDRT for OA is still practiced in Europe, where recent longitudinal studies have provided evidence of pain reduction among OA patients after LDRT [9-11]. Preliminary findings from an ongoing randomized, sham-controlled trial in Korea suggest that a single 3-Gy course of LDRT may improve knee OA symptoms at four months [12,13]. There is a clinical need for intermediate treatment options for patients who experience inadequate symptom relief from weak pain medications and injection-based therapies but do not want to pursue surgery. This can be accomplished by developing an arthritis clinic that brings together a multidisciplinary team focused on individualized treatment strategies. 

## Case presentation

A 72-year-old man presented to the office in October 2025 with a history of diffuse OA. His medical history included severe OA in the carpal bones; both capitates were removed, four meniscectomies in the right knee, and three meniscectomies in the left knee, but no knee replacements. The OA affected his right knee more than his left knee. Additionally, the patient had a medical history of hypertension and hypercholesterolemia, controlled with dietary modification, antihypertensive, and statin medication. His previous OA management was rofecoxib for two years until it was taken off the market in 2004 and ibuprofen 600 mg daily as needed for pain, but this only provided temporary relief. The patient continued using ibuprofen a couple of times a week as needed throughout LDRT treatment. Physical therapy was recommended; however, because there was no restricted range of motion or limitations in daily activities, it was not pursued. Total knee arthroplasty was also recommended, but the patient did not feel the OA was severe enough to warrant aggressive surgery. The patient still had residual cartilage in both knees.

**Figure 1:**
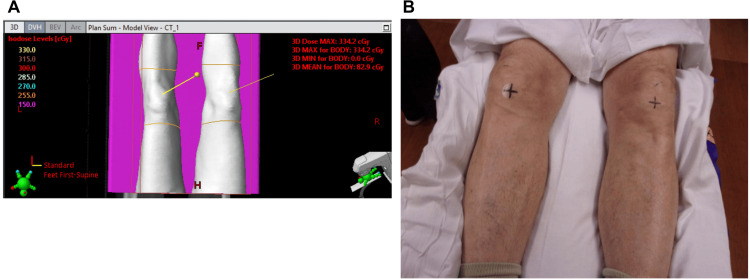
Field setup (A) Beam's eye view for left and right knee portals. (B) Setup view for left and right knee portals.

**Figure 2:**
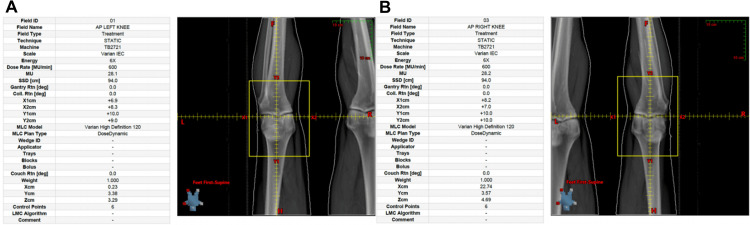
Field verification Digital radiograph of field setup for (A) left and (B) right knee portals.

**Figure 3:**
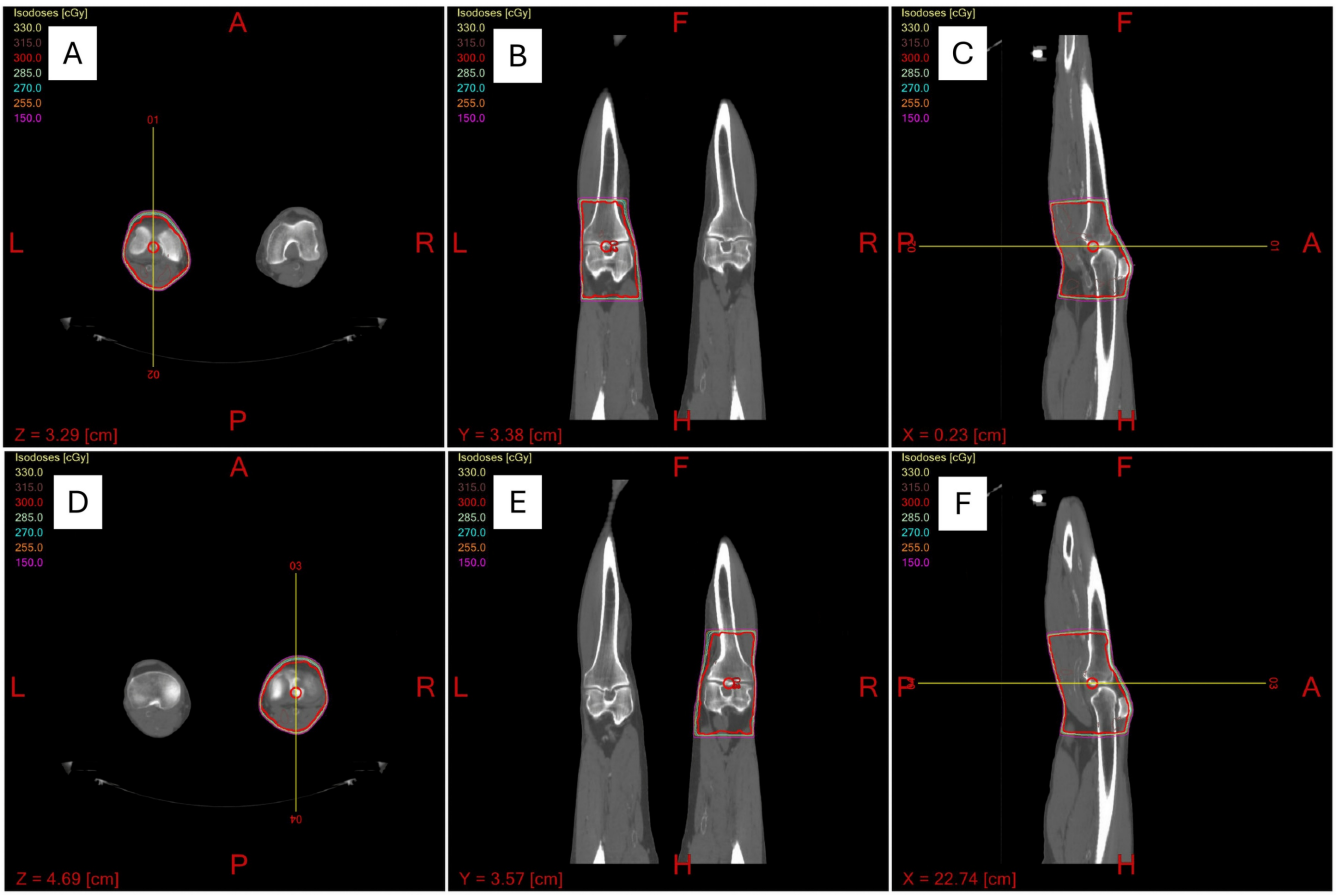
Field plan (A) Cross-sectional, (B) coronal, and (C) sagittal dosimetric plans for the left knee. (D) Cross-sectional, (E) coronal, and (F) sagittal dosimetric plans for the right knee. All these slices show the isodose lines covering 100% of the regions of interest, which include the joint space and surrounding soft tissue. There were no hot or cold spots appreciated in any of the treatment plans with symmetrical and complete dose homogeneity.

His prior pain management regimen was effective at relieving pain at rest; however, during recreational activities, his pain intensity was 7 out of 10 in the right knee and 6 out of 10 in the left knee measured on a 0-10 verbal numerical rating scale (NRS) (Table 1) [14]. On the NRS, 0 indicated no pain, and 10 indicated the worst imaginable pain. Physical examination revealed no restricted range of motion with extension or flexion of the knee. Palpation revealed tenderness around the patellofemoral joint. The patient reported limitations in recreational activities, including golf, running, and tennis. Preplanning computed tomography (CT) scans of both patellae was ordered. CT imaging revealed a lateral subluxation of the right patella and residual cartilage in both knees (Figures 1-3). Based on the imaging findings, the patient received 3 Gy/6 fractions of LDRT with a conformal treatment technique and immobilization. Both knees were treated on Monday, Wednesday, and Friday for two consecutive weeks. The treatment plan consisted of an anterior-posterior/posterior-anterior (AP/PA) approach to both the left and right knees, utilizing the field-in-field (FIF) technique to reduce inhomogeneity [15]. 6 MeV photons were prescribed in each instance. He returned to the office for a follow-up after the completion of LDRT. The patient's progress was evaluated using a verbal NRS. Two weeks post-treatment completion, the patient's pain intensity during recreational activity decreased to 3 out of 10 in the right knee and 2 out of 10 in the left knee on the NRS (Table 1). There was additional improvement in recreational activities, such as biking and running, reflecting improvement in flexibility and treatment effectiveness. Additionally, he experienced no adverse side effects from the LDRT.

**Table 1:** Patient self-reported knee pain on a NRS during recreational activities pre- and post-LDRT treatment ^1^(post-LDRT NRS pain-pre-LDRT NRS pain) ^2^(post-LDRT NRS pain-pre-LDRT NRS pain/pre-LDRT NRS pain)×100 NRS: numerical rating scale; LDRT: low-dose radiation therapy

Knee	NRS pain pre-LDRT	NRS pain post-LDRT	Absolute change in NRS pain^1^	Relative change in NRS pain^2^
Right	7	3	-4	-57.1%
Left	6	2	-4	-66.7%

## Discussion

OA is the most prevalent form of arthritis, affecting an estimated 595 million people globally in 2020, and is expected to impact 642 million by 2050 [3]. As the number of OA cases is expected to increase rapidly over the next two decades with the aging global population [2,3], there is a growing need to assess the efficacy of alternative OA treatments. LDRT has been investigated for the management of musculoskeletal disorders, such as OA, due to its anti-inflammatory effects. Macrophages and neutrophils have been shown to play major roles in the destruction and inflammation of OA joints [9]. Macrophages secrete inflammatory cytokines and metalloproteinases, while neutrophils release tumor necrosis factor-alpha (TNF-α) and neutrophil elastase, leading to progressive cartilage damage over time. LDRT can induce apoptosis in these cells, drive macrophages toward an anti-inflammatory M2 phenotype, and reduce the production of proinflammatory cytokines [4]. The case discussed here demonstrates reduced pain in patellofemoral OA and improved function during recreational activities after receiving 50 cGy per fraction (total of 3 Gy/6 fractions) to each knee three times per week for two weeks.

The Osteoarthritis Research Society International (OARSI) developed a set of responder criteria for clinical trials of OA treatment outcomes (OMERACT) based on three symptomatic domains: pain, function, and a patient's global assessment [16]. A responder is defined as an individual who has either a great improvement in pain or function (≥50% relative improvement or ≥20-unit absolute improvement on a 100-unit scale). These criteria have been used to evaluate the efficacy of various OA treatments and are being used in an ongoing randomized controlled trial of LDRT for knee OA in Korea [12,13]. The preliminary results of this trial showed that the four-month OMERACT-OARSI responder rate was significantly higher in the 3 Gy LDRT group than in the sham LDRT group (70.3% vs. 41.7%; p=0.014). The authors reported that among 114 patients with mild-to-moderate knee OA treated with a 3 Gy radiation regimen, 70% demonstrated overall improvement in pain and physical function four months post-treatment. Similarly, Niewald et al. reported improvements in pain relief after three months of 3 Gy delivered to 87 hands and 34 knees, with preserved joint structure [9]. These outcomes support the beneficial use of LDRT as a non-invasive alternative for OA management, specifically in patients with residual joint cartilage. Relative to baseline, our patient's self-reported pain had a 4 NRS unit absolute decrease in both knees with a 57.1% relative decrease in pain in the right knee and a 66.7% relative decrease in pain in the left knee, indicating a response to treatment according to the OARSI responder criteria [16]. This may suggest the efficacy of LDRT in our patient's case. Given that radiation does not promote tissue regeneration, LDRT is ideally suited for patients with persistent inflammation and preserved joint cartilage.

This case also emphasizes the need for a comprehensive approach in evaluating patients at different stages of OA and regularly reassessing their responsiveness to therapy when determining management options. The American College of Rheumatology and Arthritis Foundation provide recommendations to guide the clinical management of OA. However, the implementation of these guidelines in practice has been suboptimal, with gaps and delays between the recommended care typically provided to OA patients and the identification of which patients should be referred to specialist care [17]. The management of OA could be optimized by establishing an arthritis clinic that fosters collaboration among rheumatology, primary care, radiation oncology, and orthopedics. Once a week, rheumatologists, radiation oncologists, and orthopedic physicians would collaborate in a clinic within the hospital where they currently practice to deliver multidisciplinary care for patients with refractory OA. OA is typically diagnosed through rheumatologic assessment, using inflammatory markers, and symptoms such as short-lived morning stiffness, increased pain with use, swelling, warmth, crepitus, and decreased range of motion [1]. Radiography may be used to assess the structural changes in OA using the Kellgren-Lawrence OA grading system [18]. Magnetic resonance imaging (MRI) or CT scans can assess the articular cartilage to determine suitability for LDRT. Based on these preliminary assessments, patients will begin with interventions targeting modifiable factors, such as weight loss, muscle strengthening, and knee braces, and then receive pharmacological treatment when appropriate.

Despite evidence supporting non-pharmacological interventions for OA, such as weight loss and exercise, their implementation in practice remains difficult [19]. Continuing to follow up with the patient to review pain scores, function, symptoms, treatment side effects, and outcomes can help prevent treatment delays and improve patient outcomes. A collaborative, multidisciplinary arthritis clinic also ensures seamless information sharing between the patient and the specialist team, reducing the likelihood of overlooking evolving symptoms and exploring other management options. Future studies could evaluate if there is increased efficacy of LDRT when paired with standard treatment measures such as weight loss, physical therapy, and NSAIDs.

This case report also has some limitations, such as reporting findings for a single patient one post-treatment follow-up at two weeks. The inclusion of longer-term follow-up at multiple time points and a comparison group would provide stronger evidence for a durable effect of LDRT on pain and range of motion in OA patients. The inclusion of patients at various stages of OA would provide information on which patient populations would benefit most from LDRT. Another limitation is that we only measured the effect of LDRT on pain with a verbal rating on an NRS. Including multidimensional functional outcome measures would provide stronger evidence of the broader impacts of LDRT. Lastly, without a comparison group, we are unable to conclude that the observed reduction in pain reported by our patient is due solely to LDRT.

## Conclusions

This case highlights the potential of LDRT for pain management in knee OA, among patients with a suboptimal response to pharmacological therapy. The results from completed and ongoing randomized controlled trials of LDRT for OA in Europe and Korea indicate that it may be an alternative treatment option. While the pain relief our patient reported post-LDRT supports previous evidence of LDRT efficacy for OA pain management, additional studies are needed to assess its effectiveness and safety alongside existing treatment methods. Specifically, pragmatic randomized controlled trials in the United States could help assess whether LDRT is effective, safe, and acceptable to patients as an OA treatment in realistic clinical settings. The overall quality of OA management is suboptimal in many countries since there is no tool available to help clinicians determine which therapies are best suited for OA patients or when a referral to specialist care is necessary. The development and evaluation of alternative treatment strategies will be essential to meet the needs of the growing population of patients with OA.
